# 3-[4-(Trifluoro­meth­yl)phen­yl]propanoic acid

**DOI:** 10.1107/S1600536809010125

**Published:** 2009-03-25

**Authors:** Jian-Ning Guan, Xiang-Jun Kong, Bin Xu, Jin-Hua Liang, Na Song

**Affiliations:** a College of Science, Nanjing University of Technology, Xinmofan Road No. 5 Nanjing, Nanjing 210009, People’s Republic of China

## Abstract

In crystal of the the title compound, C_10_H_9_F_3_O_2_, inversion dimers linked by pairs of O—H⋯O hydrogen bonds occur.

## Related literature

For bond-length data, see: Allen *et al.* (1987 ▶). For related literature on acid derivatives, see: Battistuzzi *et al.* (2003 ▶); Feuerstein *et al.* (2001 ▶, 2003 ▶); Johnson & Wen (1981 ▶); Shoda & Kuriyama (2003 ▶); Yamanouchi & Yamane (1988 ▶).
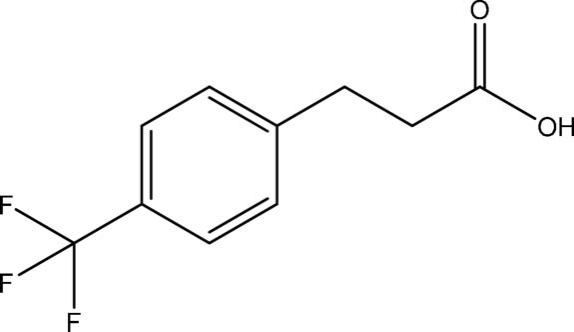


## Experimental

### 

#### Crystal data

C_10_H_9_F_3_O_2_
*M* *_r_* = 218.17
Triclinic, 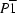

*a* = 7.9028 (19) Å
*b* = 8.288 (2) Å
*c* = 9.238 (3) Å
α = 63.381 (15)°
β = 85.20 (3)°
γ = 65.70 (2)°
*V* = 489.2 (2) Å^3^
*Z* = 2
Mo *K*α radiation
μ = 0.14 mm^−1^
*T* = 298 K
0.20 × 0.10 × 0.10 mm

#### Data collection

Enraf–Nonius CAD-4 diffractometer
Absorption correction: ψ scan (North *et al.*, 1968 ▶) *T*_min_ = 0.973, *T*_max_ = 0.986
1894 measured reflections
1754 independent reflections
874 reflections with *I* > 2σ(*I*)
*R*_int_ = 0.017
3 standard reflections every 200 reflections intensity decay: 1%

#### Refinement

*R*[*F* ^2^ > 2σ(*F* ^2^)] = 0.068
*wR*(*F* ^2^) = 0.178
*S* = 1.00
1754 reflections
128 parameters
H-atom parameters constrained
Δρ_max_ = 0.19 e Å^−3^
Δρ_min_ = −0.17 e Å^−3^

### 

Data collection: *CAD-4 EXPRESS* (Enraf–Nonius, 1994 ▶); cell refinement: *CAD-4 EXPRESS*; data reduction: *XCAD4* (Harms & Wocadlo, 1995 ▶); program(s) used to solve structure: *SHELXS97* (Sheldrick, 2008 ▶); program(s) used to refine structure: *SHELXL97* (Sheldrick, 2008 ▶); molecular graphics: *SHELXL97*; software used to prepare material for publication: *SHELXL97*.

## Supplementary Material

Crystal structure: contains datablocks global, I. DOI: 10.1107/S1600536809010125/at2744sup1.cif

Structure factors: contains datablocks I. DOI: 10.1107/S1600536809010125/at2744Isup2.hkl

Additional supplementary materials:  crystallographic information; 3D view; checkCIF report

## Figures and Tables

**Table 1:** Hydrogen-bond geometry (Å, °)

*D*—H⋯*A*	*D*—H	H⋯*A*	*D*⋯*A*	*D*—H⋯*A*
O1—H1*A*⋯O2^i^	0.82	1.87	2.687 (4)	174

Symmetry code: (i) 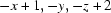.

**Fig. 1.**
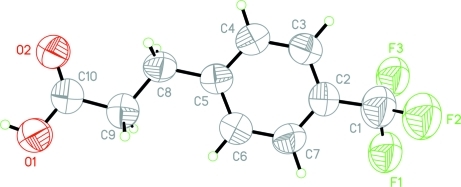
The molecular structure of (I), showing the atom-numbering scheme and displacement ellipsoids at the 30% probability level.

**Fig. 2.**
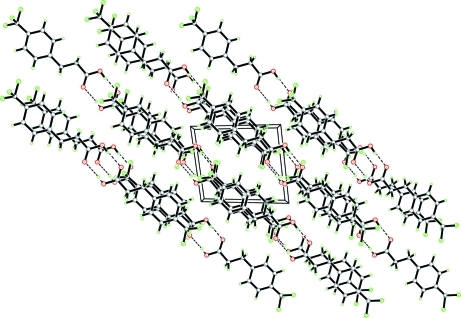
View of the packing and hydrogen bonding interactions of (I).

## supplementary crystallographic information

### Comment

Some derivatives of acids is important chemical material (Battistuzzi *et al.*, 2003; Feuerstein *et al.*, 2003, 2001; Johnson & Wen, 1981; Yamanouchi & Yamane 1988; Shoda & Kuriyama, 2003). We report here the crystal structure of the title compound, (I). The molecular structure of (I) is shown in Fig. 1, and the selected geometric parameters are given in Table 1. The bond lengths and angles (Table 1) are within normal ranges (Allen *et al.*, 1987).

In the crystal of the title compound, there is an intermolecular O—H···O hydrogen bond which may be effective to the stabilization of the crystal.

### Experimental

2,2-Dimethyl-5-(4-trifluoromethyl-benzyl)-[1,3]dioxane-4,6-dione (1 mmol), acetonitrile (25 ml) and water (0.25 ml) were mixed and subjected to microwave irradiation at 120° for 30 min (150 psi, 300 W, run time 5 min, hold time 30 min). Crude compound (I) was obtained. An X-ray grade crystal of (I) (500 mg) was grown from ethyl ether (10 ml) at room temperature.

### Refinement

H atoms were placed geometrically at the distances of C—H = 0.93–0.97 Å and O—H = 0.82 Å, and included in the refinement in riding motion approximation with *U*_iso_(H) = 1.2 or 1.5*U*_eq_ of the carrier atom.

### Crystal data

**Table:**

C_10_H_9_F_3_O_2_	*Z* = 2
*M**_r_* = 218.17	*F*(000) = 224
Triclinic, *P*1	*D*_x_ = 1.481 Mg m^−^^3^
Hall symbol: -P 1	Melting point: 379 K
*a* = 7.9028 (19) Å	Mo *K*α radiation, λ = 0.71073 Å
*b* = 8.288 (2) Å	Cell parameters from 25 reflections
*c* = 9.238 (3) Å	θ = 9–12°
α = 63.381 (15)°	µ = 0.14 mm^−^^1^
β = 85.20 (3)°	*T* = 298 K
γ = 65.70 (2)°	Yellow, colourless
*V* = 489.2 (2) Å^3^	0.20 × 0.10 × 0.10 mm

### Data collection

**Table:**

Enraf–Nonius CAD-4 diffractometer	874 reflections with *I* > 2σ(*I*)
Radiation source: fine-focus sealed tube	*R*_int_ = 0.017
graphite	θ_max_ = 25.3°, θ_min_ = 2.5°
ω/2θ scans	*h* = 0→9
Absorption correction: ψ scan (North *et al.*, 1968)	*k* = −9→9
*T*_min_ = 0.973, *T*_max_ = 0.986	*l* = −11→11
1894 measured reflections	3 standard reflections every 200 reflections
1754 independent reflections	intensity decay: 1%

### Refinement

**Table:**

Refinement on *F*^2^	Primary atom site location: structure-invariant direct methods
Least-squares matrix: full	Secondary atom site location: difference Fourier map
*R*[*F*^2^ > 2σ(*F*^2^)] = 0.068	Hydrogen site location: inferred from neighbouring sites
*wR*(*F*^2^) = 0.178	H-atom parameters constrained
*S* = 1.00	*w* = 1/[σ^2^(*F*_o_^2^) + (0.06*P*)^2^ + 0.216*P*] where *P* = (*F*_o_^2^ + 2*F*_c_^2^)/3
1754 reflections	(Δ/σ)_max_ < 0.001
128 parameters	Δρ_max_ = 0.19 e Å^−^^3^
0 restraints	Δρ_min_ = −0.17 e Å^−^^3^

### Special details

**Table:**

Geometry. All e.s.d.'s (except the e.s.d. in the dihedral angle between two l.s. planes) are estimated using the full covariance matrix. The cell e.s.d.'s are taken into account individually in the estimation of e.s.d.'s in distances, angles and torsion angles; correlations between e.s.d.'s in cell parameters are only used when they are defined by crystal symmetry. An approximate (isotropic) treatment of cell e.s.d.'s is used for estimating e.s.d.'s involving l.s. planes.
Refinement. Refinement of *F*^2^ against ALL reflections. The weighted *R*-factor *wR* and goodness of fit *S* are based on *F*^2^, conventional *R*-factors *R* are based on *F*, with *F* set to zero for negative *F*^2^. The threshold expression of *F*^2^ > σ(*F*^2^) is used only for calculating *R*-factors(gt) *etc*. and is not relevant to the choice of reflections for refinement. *R*-factors based on *F*^2^ are statistically about twice as large as those based on *F*, and *R*- factors based on ALL data will be even larger.

### Fractional atomic coordinates and isotropic or equivalent isotropic displacement parameters (Å^2^)

**Table:**

	*x*	*y*	*z*	*U*_iso_*/*U*_eq_	Occ. (<1)
O1	0.7456 (3)	−0.0616 (4)	1.0251 (3)	0.0961 (9)	
H1A	0.6554	−0.0765	1.0699	0.144*	
O2	0.5346 (4)	0.1263 (4)	0.8086 (3)	0.0888 (9)	
F1	1.5281 (8)	0.2427 (10)	0.2421 (7)	0.1096 (8)	0.429 (2)
F2	1.3838 (9)	0.5350 (11)	0.1735 (8)	0.1096 (8)	0.429 (2)
F3	1.3223 (9)	0.3992 (11)	0.0426 (8)	0.1096 (8)	0.429 (2)
F1'	1.4156 (7)	0.2852 (8)	0.1174 (6)	0.1096 (8)	0.571 (2)
F2'	1.4908 (7)	0.3848 (8)	0.2634 (6)	0.1096 (8)	0.571 (2)
F3'	1.2747 (6)	0.5871 (7)	0.0624 (6)	0.1096 (8)	0.571 (2)
C1	1.3412 (7)	0.3971 (8)	0.1896 (6)	0.1096 (8)	
C2	1.2089 (5)	0.3532 (5)	0.2963 (4)	0.0649 (9)	
C3	1.0233 (5)	0.4424 (6)	0.2356 (5)	0.0865 (12)	
H3A	0.9861	0.5349	0.1262	0.104*	
C4	0.8919 (5)	0.3987 (6)	0.3320 (5)	0.0906 (13)	
H4A	0.7682	0.4591	0.2857	0.109*	
C5	0.9377 (5)	0.2676 (5)	0.4958 (4)	0.0628 (9)	
C6	1.1224 (5)	0.1850 (7)	0.5566 (5)	0.0921 (13)	
H6A	1.1592	0.0968	0.6670	0.111*	
C7	1.2561 (5)	0.2288 (6)	0.4585 (4)	0.0827 (12)	
H7A	1.3794	0.1723	0.5046	0.099*	
C8	0.7910 (5)	0.2231 (6)	0.5981 (4)	0.0780 (11)	
H8A	0.7335	0.1714	0.5508	0.094*	
H8B	0.6948	0.3479	0.5891	0.094*	
C9	0.8489 (5)	0.0810 (5)	0.7754 (4)	0.0756 (11)	
H9A	0.9393	−0.0470	0.7859	0.091*	
H9B	0.9113	0.1283	0.8231	0.091*	
C10	0.6946 (6)	0.0527 (6)	0.8697 (5)	0.0716 (10)	

### Atomic displacement parameters (Å^2^)

**Table:**

	*U*^11^	*U*^22^	*U*^33^	*U*^12^	*U*^13^	*U*^23^
O1	0.0801 (18)	0.104 (2)	0.0801 (18)	−0.0392 (16)	0.0072 (14)	−0.0214 (16)
O2	0.0765 (18)	0.100 (2)	0.0808 (17)	−0.0409 (16)	0.0095 (14)	−0.0300 (15)
F1	0.1028 (18)	0.119 (2)	0.1119 (18)	−0.0560 (17)	0.0344 (13)	−0.0514 (15)
F2	0.1028 (18)	0.119 (2)	0.1119 (18)	−0.0560 (17)	0.0344 (13)	−0.0514 (15)
F3	0.1028 (18)	0.119 (2)	0.1119 (18)	−0.0560 (17)	0.0344 (13)	−0.0514 (15)
F1'	0.1028 (18)	0.119 (2)	0.1119 (18)	−0.0560 (17)	0.0344 (13)	−0.0514 (15)
F2'	0.1028 (18)	0.119 (2)	0.1119 (18)	−0.0560 (17)	0.0344 (13)	−0.0514 (15)
F3'	0.1028 (18)	0.119 (2)	0.1119 (18)	−0.0560 (17)	0.0344 (13)	−0.0514 (15)
C1	0.1028 (18)	0.119 (2)	0.1119 (18)	−0.0560 (17)	0.0344 (13)	−0.0514 (15)
C2	0.075 (2)	0.060 (2)	0.073 (2)	−0.032 (2)	0.0156 (18)	−0.0384 (19)
C3	0.074 (3)	0.085 (3)	0.074 (2)	−0.025 (2)	0.004 (2)	−0.021 (2)
C4	0.064 (2)	0.095 (3)	0.088 (3)	−0.025 (2)	0.007 (2)	−0.029 (2)
C5	0.067 (2)	0.064 (2)	0.067 (2)	−0.0261 (18)	0.0045 (17)	−0.0388 (18)
C6	0.080 (3)	0.108 (3)	0.071 (2)	−0.042 (3)	0.003 (2)	−0.023 (2)
C7	0.073 (2)	0.108 (3)	0.073 (2)	−0.045 (2)	0.0032 (19)	−0.039 (2)
C8	0.069 (2)	0.102 (3)	0.070 (2)	−0.039 (2)	0.0010 (18)	−0.042 (2)
C9	0.079 (2)	0.067 (2)	0.082 (2)	−0.030 (2)	0.016 (2)	−0.037 (2)
C10	0.081 (3)	0.069 (3)	0.082 (3)	−0.040 (2)	0.016 (2)	−0.042 (2)

### Geometric parameters (Å, °)

**Table:**

O1—C10	1.298 (4)	C4—C5	1.377 (5)
O1—H1A	0.8200	C4—H4A	0.9300
O2—C10	1.211 (4)	C5—C6	1.373 (5)
F1—C1	1.436 (8)	C5—C8	1.493 (5)
F2—C1	1.264 (8)	C6—C7	1.389 (5)
F3—C1	1.371 (8)	C6—H6A	0.9300
F1'—C1	1.300 (6)	C7—H7A	0.9300
F2'—C1	1.359 (6)	C8—C9	1.494 (5)
F3'—C1	1.379 (6)	C8—H8A	0.9700
C1—C2	1.420 (5)	C8—H8B	0.9700
C2—C7	1.357 (4)	C9—C10	1.475 (5)
C2—C3	1.373 (5)	C9—H9A	0.9700
C3—C4	1.367 (5)	C9—H9B	0.9700
C3—H3A	0.9300		
			
C10—O1—H1A	109.5	C3—C4—C5	121.8 (4)
F2—C1—F1'	124.1 (5)	C3—C4—H4A	119.1
F2—C1—F2'	50.9 (4)	C5—C4—H4A	119.1
F1'—C1—F2'	104.0 (5)	C6—C5—C4	116.1 (4)
F2—C1—F3	111.3 (5)	C6—C5—C8	123.3 (3)
F1'—C1—F3	40.0 (3)	C4—C5—C8	120.6 (3)
F2'—C1—F3	128.7 (5)	C5—C6—C7	122.1 (4)
F2—C1—F3'	53.5 (4)	C5—C6—H6A	119.0
F1'—C1—F3'	103.4 (5)	C7—C6—H6A	119.0
F2'—C1—F3'	102.5 (5)	C2—C7—C6	120.7 (4)
F3—C1—F3'	68.0 (4)	C2—C7—H7A	119.6
F2—C1—C2	119.6 (5)	C6—C7—H7A	119.6
F1'—C1—C2	116.3 (5)	C5—C8—C9	118.1 (3)
F2'—C1—C2	113.8 (4)	C5—C8—H8A	107.8
F3—C1—C2	115.5 (5)	C9—C8—H8A	107.8
F3'—C1—C2	115.2 (4)	C5—C8—H8B	107.8
F2—C1—F1	96.1 (5)	C9—C8—H8B	107.8
F1'—C1—F1	58.3 (4)	H8A—C8—H8B	107.1
F2'—C1—F1	50.5 (3)	C10—C9—C8	114.8 (3)
F3—C1—F1	95.9 (5)	C10—C9—H9A	108.6
F3'—C1—F1	130.2 (4)	C8—C9—H9A	108.6
C2—C1—F1	114.2 (4)	C10—C9—H9B	108.6
C7—C2—C3	117.5 (3)	C8—C9—H9B	108.6
C7—C2—C1	123.0 (4)	H9A—C9—H9B	107.5
C3—C2—C1	119.5 (4)	O2—C10—O1	122.4 (3)
C4—C3—C2	121.6 (4)	O2—C10—C9	123.5 (4)
C4—C3—H3A	119.2	O1—C10—C9	114.0 (4)
C2—C3—H3A	119.2		
			
F2—C1—C2—C7	−85.8 (7)	C2—C3—C4—C5	2.0 (7)
F1'—C1—C2—C7	92.3 (6)	C3—C4—C5—C6	0.5 (6)
F2'—C1—C2—C7	−28.5 (7)	C3—C4—C5—C8	179.9 (4)
F3—C1—C2—C7	137.0 (5)	C4—C5—C6—C7	−0.7 (6)
F3'—C1—C2—C7	−146.5 (5)	C8—C5—C6—C7	179.9 (4)
F1—C1—C2—C7	27.1 (7)	C3—C2—C7—C6	3.8 (6)
F2—C1—C2—C3	92.8 (7)	C1—C2—C7—C6	−177.6 (4)
F1'—C1—C2—C3	−89.2 (6)	C5—C6—C7—C2	−1.5 (7)
F2'—C1—C2—C3	150.0 (5)	C6—C5—C8—C9	−0.1 (5)
F3—C1—C2—C3	−44.4 (7)	C4—C5—C8—C9	−179.5 (4)
F3'—C1—C2—C3	32.0 (7)	C5—C8—C9—C10	177.0 (3)
F1—C1—C2—C3	−154.3 (5)	C8—C9—C10—O2	5.0 (5)
C7—C2—C3—C4	−4.1 (6)	C8—C9—C10—O1	−176.0 (3)
C1—C2—C3—C4	177.2 (4)		

### Hydrogen-bond geometry (Å, °)

**Table:**

*D*—H···*A*	*D*—H	H···*A*	*D*···*A*	*D*—H···*A*
O1—H1A···O2^i^	0.82	1.87	2.687 (4)	174

Symmetry codes: (i) −*x*+1, −*y*, −*z*+2.
